# HSP27 Inhibits Homocysteine-Induced Endothelial Apoptosis by Modulation of ROS Production and Mitochondrial Caspase-Dependent Apoptotic Pathway

**DOI:** 10.1155/2016/4847874

**Published:** 2016-04-17

**Authors:** Xin Tian, Lei Zhao, Xianjing Song, Youyou Yan, Ning Liu, Tianyi Li, Bingdi Yan, Bin Liu

**Affiliations:** ^1^Department of Cardiology, The Second Hospital of Jilin University, 218 Ziqiang Street, Changchun 130041, China; ^2^Department of Respiratory Medicine, The Second Hospital of Jilin University, 218 Ziqiang Street, Changchun 130041, China

## Abstract

*Objectives*. Elevated plasma homocysteine (Hcy) could lead to endothelial dysfunction and is viewed as an independent risk factor for atherosclerosis. Heat shock protein 27 (HSP27), a small heat shock protein, is reported to exert protective effect against atherosclerosis. This study aims to investigate the protective effect of HSP27 against Hcy-induced endothelial cell apoptosis in human umbilical vein endothelial cells (HUVECs) and to determine the underlying mechanisms.* Methods*. Apoptosis, reactive oxygen species (ROS), and mitochondrial membrane potential (MMP) of normal or HSP27-overexpressing HUVECs in the presence of Hcy were analyzed by flow cytometry. The mRNA and protein expression levels were measured by quantitative real-time polymerase chain reaction (qRT-PCR) and western blot.* Results*. We found that Hcy could induce cell apoptosis with corresponding decrease of nitric oxide (NO) level, increase of endothelin-1 (ET-1), intracellular adhesion molecule-1 (ICAM-1), vascular cellular adhesion molecule-1 (VCAM-1), and monocyte chemoattractant protein-1 (MCP-1) levels, elevation of ROS, and dissipation of MMP. In addition, HSP27 could protect the cell against Hcy-induced apoptosis and inhibit the effect of Hcy on HUVECs. Furthermore, HSP27 could increase the ratio of Bcl-2/Bax and inhibit caspase-3 activity.* Conclusions*. Therefore, we concluded that HSP27 played a protective role against Hcy-induced endothelial apoptosis through modulation of ROS production and the mitochondrial caspase-dependent apoptotic pathway.

## 1. Introduction

Atherosclerosis, a chronic immunoinflammatory disease, is the most common underlying cause of cardiovascular and peripheral vascular diseases [1, 2]. Elevation of the plasma level of Hcy is one of the recognized independent risk factors for the development of atherosclerosis [3–5]. Evidence shows that the elevation of plasma Hcy can lead to endothelial dysfunction [6, 7], an early marker for atherosclerosis [8, 9]. It was reported that endothelial cell apoptosis contributed to endothelial dysfunction and destabilization of atherosclerotic plaques and thrombosis [10].

HSP27, a member of the small heat shock protein family, is involved in a wide variety of cellular processes such as apoptosis [11], inflammation [12], cell migration [13], and maintenance of arterial wall homeostasis [14]. HSP27 in atherosclerotic plaques diminishes with progression of the stage of the pathology [15, 16] and is thereby viewed as a potential biomarker [14]. It has also been shown that HSP27 can protect cells from apoptosis induced by various stimuli in vivo and in vitro [17–20]. This cytoprotective effect of HSP27 is associated with decrease in ROS [21] and increase in glutathione [22] during oxidative stress. In addition, HSP27 acts both upstream and downstream of cytochrome c release in a stimulus-dependent manner [23] and inhibits caspase-3 activity through interaction with its inactive form, procaspase-3 [24]. However, modulation in HSP27 expression toward endothelial protection against Hcy treatment has not been investigated.

The objectives of this study were to determine the effect of HSP27 on Hcy-induced endothelial cell apoptosis and to elucidate the possible protective mechanism of HSP27 against endothelial dysfunction.

## 2. Materials and Methods 

### 2.1. Materials

Rh123 mitochondrial specific fluorescent dye was purchased from Sigma. Reactive Oxygen Species Assay Kit was purchased from Beyotime Company (Shanghai, China). Dulbecco's modified Eagle's medium (DMEM) and fetal bovine serum (FBS) were purchased from Gibco-Invitrogen. D,L-Hcy was purchased from Sigma (Aldrich, America). BCA Protein Assay Kit and annexin V-FITC Apoptosis Detection Kit were purchased from Keygen Company (Nanjing, China). Polyclonal antibodies against *β*-actin and horseradish peroxidase-conjugated secondary antibodies (goat-anti-rabbit, mouse) were purchased from Beyotime Biotechnology (Shanghai, China). Antibody for HSP27 was obtained from Abcam. Polyclonal antibodies against Bax, Bcl-2, caspase-3, and PARP were purchased from Cell Signaling Technology, Inc. (Danvers, MA, USA). Western blot detection kit was purchased from Millipore (Billerica, USA).

### 2.2. Cell Culture

HUVECs were maintained in DMEM supplemented with 10% FBS at 37°C in a humidified atmosphere with 5% CO_2_ and 95% air.

### 2.3. Measurement of HSP27 Expression in Hcy-Treated HUVECs

HUVECs were incubated in 100 mm plates and treated with Hcy at the concentrations of 0 mM, 5 mM, and 10 mM for 24 h. The cells were then harvested and HSP27 expression was measured by western blot.

### 2.4. Cell Transfection

HUVECs were transfected with either empty pEX-4 or HSP27-pEX-4 plasmid (GenePharma, Shanghai, China). The plasmids contained green fluorescent protein (GFP) and the neomycin resistance gene. UVECs were seeded in six-well plate with 40–60% confluence 20 h before transfection and the plasmids were transfected by Lipofectamine® 2000 (Invitrogen, Guangzhou, China) according to the user's protocol. Transfected cells were screened using 0.7 mg/mL G418 (Sigma-Aldrich, America). After 2 weeks, single G418-resistant cell clones were seeded and grown in a 96-well plate. HSP27 expression was detected using fluorescence microscopy, qRT-PCR, and western blot. HUVECs transfected with an empty plasmid were labeled as Neo while those transfected with plasmid coding HSP27 were labeled as Hsp27. These cells were prepared for subsequent experiments.

### 2.5. Measurement of NO Concentration in the Medium

Cells were incubated in six-well plates and treated with 10 mM Hcy for 24 h. The medium was collected and the total NO production was measured using a Total Nitric Oxide Assay Kit (Beyotime Biotechnology, Shanghai, China).

### 2.6. Apoptosis Analysis by Flow Cytometry

After treatment, cells were detached from the culture dishes with 0.25% trypsin and were collected by centrifugation. After washing twice with ice-cold PBS, the cells were resuspended in binding buffer containing annexin V-FITC and propidium iodide (PI) according to the manufacturer's instructions. Cells were then sorted using flow cytometry.

### 2.7. ROS Determination by Flow Cytometry

Cells were stained with 2′,7′-dichlorofluorescein diacetate (DCFH-DA). Cells were plated in six-well plate and then treated with 10 mM Hcy for another 24 h. After treatment, cells were incubated with 10 *μ*M DCFH-DA in the dark for 30 min. Finally, the cells were analyzed for DCF fluorescence by flow cytometry.

### 2.8. MMP Determination by Flow Cytometry

Cells were plated on six-well plates and then treated with 10 mM Hcy for another 24 h. Afterwards, the cells were incubated with Rhodamine 123 (10 *μ*M) at 37°C in the dark for 20 min. After filtration, the samples were analyzed by flow cytometry.

### 2.9. qRT-PCR Analysis

Total RNA was isolated using TRIzol in a sterile, RNase-free environment. Reverse transcription was done using the RevertAid Reverse Transcriptase (Thermo Scientific); the reaction was performed at 37°C for 1 h. The qRT-PCR was performed using Maxima SYBR Green qPCR Master Mix (2x) (Thermo Scientific). The reaction system contained 12.5 *μ*L of Maxima SYBR Green qPCR Master Mix (2x), 1 *μ*L of primer, 1 *μ*L of cDNA, and 10.5 *μ*L of ddH_2_O. We used Roche LightCycler 480 II System to perform qRT-PCR. The procedure was as follows: pretreatment for 1 cycle at 95°C for 2 min; initial denaturation for 1 cycle at 95°C for 30 s; 40 cycles of 95°C for 15 s and 60°C for 60 s; and melt curve stage. Fold changes in target gene expression between treatments and controls were determined using the 2^−ΔΔCt^ method, normalizing to 18S RNA expression as an internal reference. All analyses were performed in six independent experiments and were performed in triplicate. The primers used were 5′-CGGACATCTAAGGGCATCACAG-3′ (sense) and 5′-GGACACGGACAGGATTGACA-3′ (antisense) for 18S; 5′-ACGCAGTCCAACGAGATCA-3′ (sense) and 5′-CTTTACTTGGCGGCAGTCTC-3′ (antisense) for HSP27; 5′-TCTCTGCTGTTTGTGGCTTG-3′ (sense) and 5′-GGACTGGGAGTGGGTTTCTC-3′ (antisense) for ET-1; 5′-CTCAGCCAGATGCAATCAAT-3′ (sense) and 5′-GCTTCTTTGGGACACTTGCT-3′ (antisense) for MCP-1; 5′-AGCTTCTCCTGCTCTGCAAC-3′ (sense) and 5′-GACAATCCCTCTCGTCCAGT-3′ (antisense) for ICAM-1; and 5′-ACACACAGGTGGGACACAAA-3′ (sense) and 5′-AGGCTCCAAGGATCACGAC-3′ (antisense) for VCAM-1.

### 2.10. Western Blot

Cellular proteins were analyzed using western blot. Cells were collected after each experiment and lysed with RIPA buffer followed by ultrasound sonication in an ice bath. The supernatant fluids were collected after centrifugation at 13,000 g for 5 min. The BCA Protein Assay Kit was used to measure the protein concentrations. Gel electrophoresis was done using 10% SDS-PAGE gel and transferred to a 0.45 *μ*m polyvinylidene fluoride membrane (Amersham Biosciences, Piscataway, NJ). The membranes were soaked in blocking buffer (5% skimmed milk) for 1 h. Membranes were then incubated overnight at 4°C with relevant antibodies, followed by horseradish peroxidase-conjugated secondary antibodies, and enhanced chemiluminescence (ECL) detection. Gel-Pro Analyzer was used to extract valuable qualitative and quantitative information from electrophoretic gels and western blot.

### 2.11. Statistical Analysis

Data have been expressed as mean ± SD from at least three different experiments. Comparisons were made using a one-way ANOVA followed by Dunnett's test. Statistically significant results were indicated by *P* < 0.05.

## 3. Results

### 3.1. Hcy Increased HSP27 Expression in HUVECs

**Figure 1:**
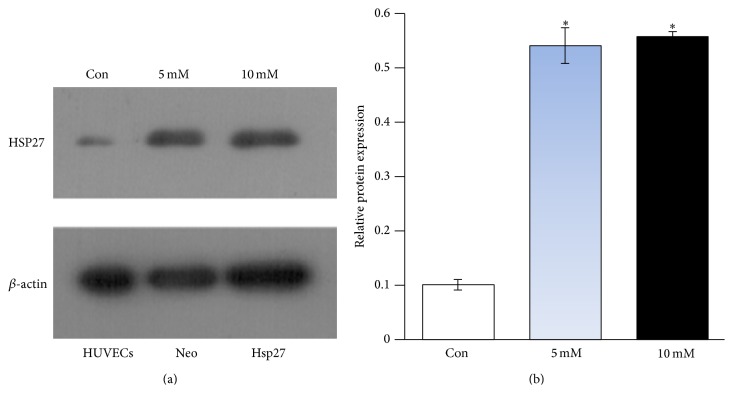
Hcy increased HSP27 expression in HUVECs. (a) Western blot analyses of the HSP27 protein levels in Hcy-treated HUVECs. Cells were treated with the indicated concentration of Hcy for 24 h; then, they were harvested and whole-cell extracts were prepared and probed for HSP27. (b) The relative protein levels were normalized to *β*-actin. Data are expressed as mean ± SD of three independent experiments. ^*∗*^ *P* < 0.05 compared with the control.

Hcy is known to induce oxidative stress, which could increase the expression of heat shock proteins such as HSP27 [25]. Therefore, we examined the effect of Hcy on the expression of HSP27 in HUVECs and found that the protein level of HSP27 was upregulated in HUVECs after treatment with Hcy (Figures 1(a) and 1(b)).

### 3.2. Construction of the HSP27-Overexpressing Cell Line

**Figure 2:**
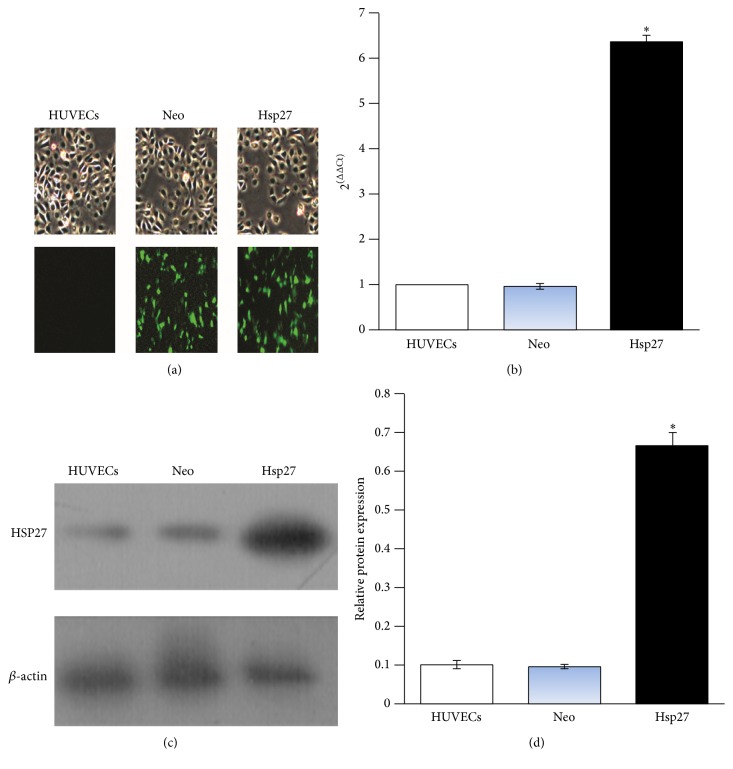
Stable HSP27 overexpressing HUVEC line (Hsp27). The pEX-4 or HSP27-pEX-4 plasmid was inserted in HUVECs. After 2 weeks of G418 selection, high expression of HSP27 as compared to that in parental HUVECs was detected in cells by fluorescence microscopy, qRT-PCR, and western blot analyses. (a) Images of HUVECs, Neo, and Hsp27 cells obtained by fluorescence microscopy (4x original magnification); the green fluorescence represented GFP. (b) Real-time RT-PCR analysis of HSP27 mRNA expression in cell lines. Total RNA was prepared and quantified using specific primers. (c) HSP27 protein expression by western blotting. (d) The relative protein levels were normalized to *β*-actin. Data are expressed as mean ± SD of three independent experiments. ^*∗*^ *P* < 0.05 compared with the Neo group.

The functions of HSP27 have not been thoroughly investigated; accumulating reports have shown that it plays an important role in cytoprotection. To investigate the functions of HSP27 in HUVECs exposed to Hcy, stable HUVECs overexpressing HSP27 were constructed. Fluorescence microscopy images of HUVECs, Neo, and HSP27 cells were shown in Figure 2(a). The data for mRNA and protein levels of HSP27 were confirmed by qRT-PCR and western blot (Figures 2(b), 2(c), and 2(d)).

### 3.3. Effects of HSP27 on NO Production and Endothelial Molecule Expression

**Figure 3:**
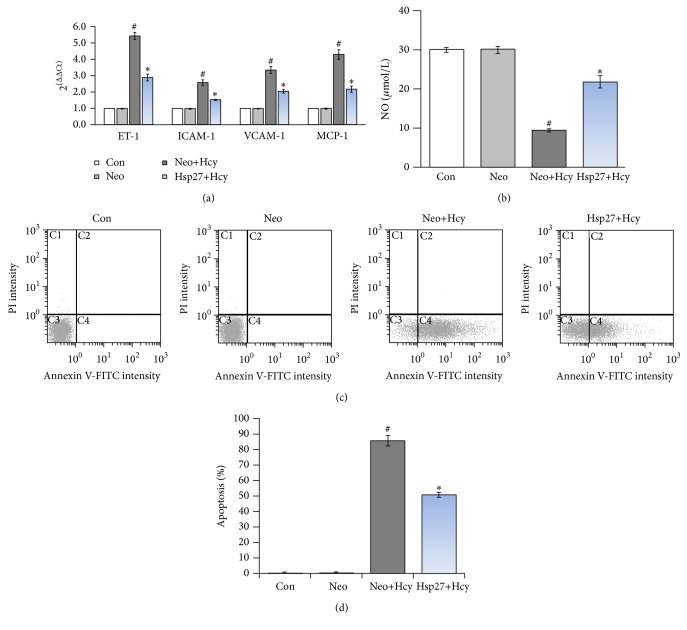
Effect of HSP27 on Hcy-induced endothelial dysfunction and apoptosis (a). Real-time RT-PCR analysis of ET-1, ICAM-1, VCAM-1, and MCP-1 mRNA expression in HUVECs. After cells were treated with 10 mM Hcy for 24 h or were not treated, total RNA was prepared and quantified using specific primers. (b) HSP27 weakened the Hcy-mediated NO decrease in HUVECs. (c) and (d) Cells that were treated with 10 mM Hcy or were not treated were analyzed after 24 h by flow cytometry. The number represents the percentages of apoptotic cells in each condition. Data are expressed as mean ± SD of three independent experiments. Columns not sharing the same superscript letter differ significantly: ^*∗*^ *P* < 0.05 compared with the Neo+Hcy group; ^#^ *P* < 0.05 compared with the Neo group.

The common feature of endothelial dysfunction is a decrease in the amount of bioavailable NO; the production of NO can be a marker of endothelial dysfunction [26]. After treatment with Hcy, the level of NO in the culture medium of the Hsp27+Hcy group was significantly higher than that for the Neo+Hcy group (Figure 3(b)). ET-1, which is synthesized predominantly by vascular endothelial cells, is the most potent vessel constrictor and vascular modulator. The expression of ET-1 increases when endothelial dysfunction occurs [27]. Endothelial cells express adhesion and chemoattractant molecules like ICAM-1, VCAM-1, and MCP-1, which are particularly implicated in vascular inflammation in atherogenic processes [28, 29]. When endothelial dysfunction occurs, the expression of ICAM-1, VCAM-1, and MCP-1 in mRNA is upregulated [30, 31]. We found that the mRNA levels of ET-1, ICAM-1, VCAM-1, and MCP-1 were higher in the Hsp27+Hcy group than the Neo+Hcy group (Figure 3(a)).

### 3.4. Protective Effect of HSP27 on Hcy-Induced Apoptosis

To investigate the effect of HSP27 on Hcy-induced apoptosis, we treated the cells with or without 10 mM Hcy for 24 h and the percentages of cells undergoing apoptosis were determined by flow cytometry analysis after staining with annexin V-FITC and PI. Compared to the Neo+Hcy group, the apoptosis rate in the Hsp27+Hcy group was decreased from 88.85% to 50.93% (Figures 3(c) and 3(d)).

### 3.5. HSP27 Attenuated Hcy-Mediated ROS Generation and MMP Reduction

**Figure 4:**
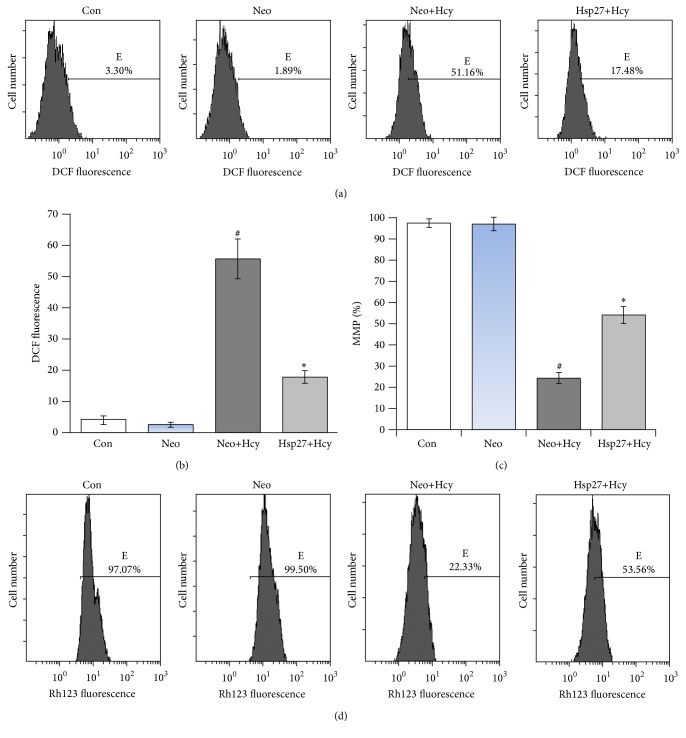
HSP27 resisted Hcy-induced elevation in ROS and MMP depletion. (a) ROS in cells that were treated with 10 mM Hcy or were not treated were analyzed after 24 h by flow cytometry. (b) and (c) Data are expressed as mean ± SD of three independent experiments. Columns not sharing the same superscript letter differ significantly: ^*∗*^ *P* < 0.05 compared with Neo+Hcy group; ^#^ *P* < 0.05 compared with the Neo group. (d) Cells were treated with 10 mM Hcy for 24 h or were not treated; then, they were collected and stained with Rh123 for flow cytometric analysis. The values indicated the percentage of Rh123 fluorescence on cells after the treatment.

ROS is a mediator of intracellular signals and plays an important role in causing apoptotic cell death [32]. A significant (*P* < 0.05) increase in the intracellular ROS level was observed in Hcy-exposed Neo cells while Hsp27 cells exhibited only ~17.48% increase (Figures 4(a) and 4(b)). Meanwhile, a 77.17% decrease of MMP was found in Hcy-exposed Neo cells, while Hsp27 cells exhibited only 46.44% decrease (Figures 4(c) and 4(d)). The data showed that HSP27 had a protective effect on Hcy-induced apoptosis and MMP disruption in HUVECs.

### 3.6. Effects of HSP27 on the Expression of Apoptosis Regulators Induced by Hcy

**Figure 5:**
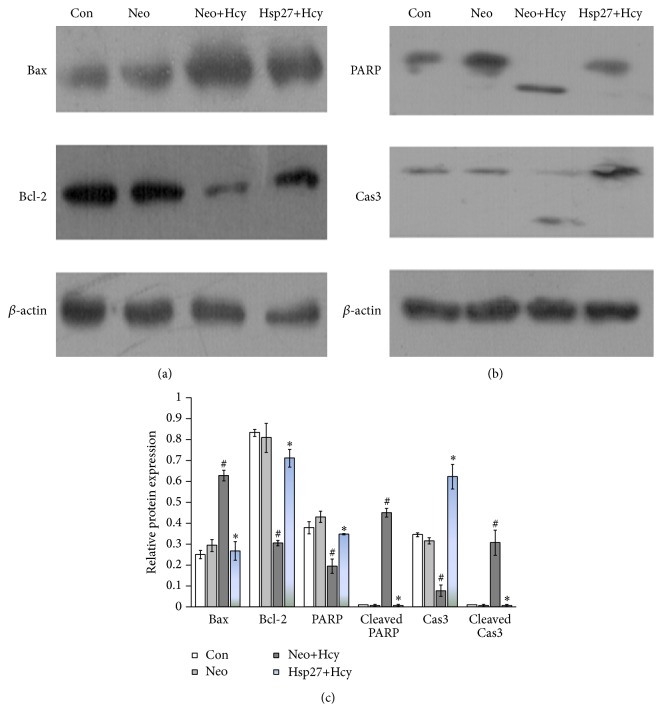
HSP27 attenuated the effect of Hcy on the expression of apoptosis regulators. Cells were treated with 10 mM Hcy for 24 h or were not treated. (a) and (b) Whole-cell extracts were prepared and probed for Bcl-2, Bax, caspase-3, and PARP by western blot analysis and quantified by the Gel-Pro Analyzer software. (c) The protein levels were normalized relative to those of *β*-actin, which was used as a loading control. Data are expressed as mean ± SD of three independent experiments. Columns not sharing the same superscript letter differ significantly: ^*∗*^ *P* < 0.05 compared with the Neo+Hcy group; ^#^ *P* < 0.05 compared with the Neo group.

The Bcl-2 protein family, a large family of apoptosis-regulating proteins, modulates the mitochondrial pathway [33]. To further characterize the function of HSP27 in Hcy-induced apoptosis, we examined the impact of HSP27 on levels of Bcl-2 family proteins in Hcy-treated HUVECs by using western blot. After treatment with Hcy, the rate of Bcl-2/Bax was increased in the Hsp27+Hcy group compared to Neo+Hcy group (Figures 5(a) and 5(c)). In addition, caspase-3 cleavage and PARP cleavage both decreased in the Hsp27+Hcy group compared to the Neo+Hcy group (Figures 5(b) and 5(c)).

## 4. Discussion

Increased plasma level of Hcy is an independent risk factor for the development of atherosclerosis [3–5]. Evidence shows that the elevation of plasma Hcy can lead to endothelial dysfunction [34, 35]. Hsp27, as a cytoprotector, has been reported to be a potent antiapoptotic molecule [11, 36, 37]. In order to investigate the role of HSP27 in endothelial cells, we tested the expression of HSP27 in Hcy-treated HUVECs. We found that Hcy treatment promoted HSP27 expression in HUVECs, while overexpression of HSP27 decreased Hcy-induced endothelial apoptosis. This may indicate that HSP27 regulated cell apoptosis in a negative feedback form.

NO is a crucial mediator in endothelial vasodilator function. Abnormal NO production in the vascular endothelium results in endothelial dysfunction, which is a prelude to atherosclerosis [38]. In the vascular system, the bioavailability of NO can be impaired by various mechanisms such as decrease in NO production due to eNOS and enhanced NO breakdown during oxidative stress. NO reacts with superoxide to form peroxynitrite. Peroxynitrite changes the redox state of the vessel wall, which might stimulate increased expression of VCAM-1, ICAM-1, E-selectin, and MCP-1 in the endothelial cells [26]. In this study, Hcy inhibited NO synthesis while HSP27 rescued NO synthesis, thereby improving endothelial function. ET-1, a potent vasoconstrictor, is primarily produced by vascular endothelial cells. ET-1 and NO are natural counterparts in vascular function. ET-1 modulates endothelial function and is upregulated after endothelial dysfunction [27, 39]. Endothelial cells also express adhesion and chemoattractant molecules such as MCP-1, VCAM-1, and ICAM-1, which recruit inflammatory monocytes into the vascular wall and initiate atherosclerosis [29, 40]. When endothelial dysfunction occurs, the mRNA and protein expression of ICAM-1, VCAM-1, and MCP-1 increases [26, 41, 42]. The results showed that Hcy increased the mRNA levels of ET-1, ICAM-1, VCAM-1, and MCP-1, and these effects could be attenuated further by HSP27. These results demonstrated that HSP27 rescued endothelial dysfunction after exposure to Hcy.

It has been reported that oxidative stress plays an important role in the pathogenesis of endothelial dysfunction and atherosclerosis [43, 44]. Elevated amounts of intracellular ROS can induce oxidative stress, loss of cell function, and cell apoptosis [45]. It has been shown that MMP loss, an early event, is directly associated with apoptosis [46]. As MMP decreases, the mitochondrial permeability transit pores (PTPs) open and cytochrome c and other proapoptotic molecules are released from the intermembrane space to the cytosol. In this study, HUVECs overexpressing HSP27 resisted intracellular ROS level elevation and MMP depletion after exposure to Hcy. Considering these results, it is evident that HSP27 could inhibit Hcy-induced endothelial apoptosis by decreasing intracellular ROS level and increasing MMP.

Bcl-2 family proteins, which include antiapoptotic proteins and proapoptotic proteins such as Bcl-2 and Bax, govern the mitochondria-dependent apoptosis pathway [33]. The results of this study showed that the expressions of Bax were increased and Bcl-2 was decreased in Hcy-treated cells, while HSP27 weakened this response. Caspase-3 has been considered as a central component of the proteolytic cascade during apoptosis and can cleave some nuclear proteins such as PARP, thereby leading to a typical apoptotic DNA fragmentation [47]. In this study, HSP27 decreased the expression of cleaved PARP and cleaved caspase-3 after treatment with Hcy. These results showed that HSP27 inhibited Hcy-induced endothelial apoptosis by modulating the mitochondrial caspase-dependent apoptotic pathway.

In conclusion, HSP27 protected against Hcy-induced endothelial apoptosis, possibly by inducing cellular ROS production and inhibiting the mitochondrial caspase-dependent apoptotic pathway. The effects of HSP27 on endothelial molecule expression and NO production represent a novel mechanism by which HSP27 modulates vascular homeostasis and may serve as a new approach for prevention of atherosclerosis. However, further research on the important role of HSP27 in protecting endothelial cells from apoptosis in vivo is warranted.
